# Cholesterol Crystal-Mediated Inflammation Is Driven by Plasma Membrane Destabilization

**DOI:** 10.3389/fimmu.2018.01163

**Published:** 2018-05-29

**Authors:** Fei Shu, Jiahuan Chen, Xiaojie Ma, Yunzhou Fan, Li Yu, Wencheng Zheng, Matthias W. Amrein, Tie Xia, Yan Shi

**Affiliations:** ^1^Peking University-Tsinghua University-National Institute of Biological Sciences Joint Graduate Program, School of Life Sciences, Peking University, Beijing, China; ^2^Department of Basic Medical Sciences, Center for Life Sciences, Institute for Immunology, Beijing Key Lab for Immunological Research on Chronic Diseases, Tsinghua University, Beijing, China; ^3^Department of Chemical Biology, School of Pharmaceutical Sciences, Peking University, Beijing, China; ^4^Department of Cell Biology, Snyder Institute, University of Calgary, Calgary, AB, Canada; ^5^Department of Anatomy, Snyder Institute, University of Calgary, Calgary, AB, Canada; ^6^Department of Microbiology, Immunology and Infectious Diseases, Snyder Institute, University of Calgary, Calgary, AB, Canada

**Keywords:** cholesterol crystals, cell death, inflammation, membrane rupture, signal free

## Abstract

Atherosclerosis is driven by an inflammatory milieu in the walls of artery vessels. Initiated early in life, it progresses to plaque formation and form cell accumulation. A culprit in this cascade is the deposition of cholesterol crystals (CC). The involvement of smaller crystals in the early stage of atherosclerotic changes may be critical to the long-term pathological development. How these small crystals initiate the pro-inflammatory events is under study. We report here an unexpected mechanism that microscopic CC interact with cellular membrane in a phagocytosis-independent manner. The binding of these crystals extracts cholesterol from the cell surface. This process causes a sudden catastrophic rupture of plasma membrane and necrosis of the bound cells independent of any known cell death-inducing pathways, releasing inflammatory agents associated with the necrotic cell death. Our results, therefore, reveal a biophysical aspect of CC in potentially mediating the inflammatory progress in atherosclerosis.

## Introduction

The accumulation of cholesterol crystals (CC) in atherosclerotic lesions reflects the imbalance of cholesterol homeostasis. LDL transports esterified cholesterol from the liver to artery walls *via* LDL receptor. The esterified cholesterol is then deposited in the subintima and becomes readily accessible to macrophages and muscle cells. Ester hydrolases in these cells convert the esterified cholesterol into its free form, leading to the crystal formation (1). This is countered by the reverse transport mediated by HDL (2). At late stages of development, large amounts of clinical data suggest that the volume expansion associated with cholesterol solidification creates a rupture force exerted on the fibrous cap of the plaques (3, 4). Thromboembolism may thus result.

The accumulation of CC starts early in young animals. Due to their minuscule sizes and limitations of standard microscopy techniques, direct visualization has not been easy. With refinements of preparation protocols (5, 6) and development of label-free Raman scattering imaging technology (7), the presence of the small crystals became detectable. Those improved detections strongly suggest their involvement in much of the initial atherosclerotic development. In fact, cholesterol-lowering treatment used early is protective but is ineffective in the later stages (8). Mechanistically, the presence of CC in the early pathogenesis is considered to be a significant contributor to local inflammation (9). It has been suggested that NLRP3 inflammasome was critically involved in the plaque formation (5, 10). However, other reports failed to recapture this association (11, 12). On the other hand, several groups have found that IL-1α (12) and the complement system (13) are activated by CC. Therefore, how CC-mediated inflammation contributes to the early vascular damage is still not well understood. One suspected but ill-defined aspect of CC-mediated cytotoxicity is their ability to damage the plasma membrane (14).

In studying phagocytosis of particulate structures, we made a surprising finding that unlike phagocytosis of most solid structures, the adhesion force between macrophages and CC does not rely on Syk kinase, a common signaling intermediate in phagocytosis. The attachment strength between CC and plasma membrane is a function of the cholesterol in the latter. Furthermore, the binding of CC causes the transfer of membrane cholesterol to the crystal, leading to necrosis in macrophages. This physical damage-induced cell death is pro-inflammatory yet independent of common cell death induction pathways, including NLRP3 inflammasome, mixed lineage kinase domain-like kinase (MLKL) (15), gasdermin D (GSDMD) (16), Caspase 1/8, Ca^2+^ signaling (17), and calpain-mediated cellular damage (18). In fact, this membrane destabilization can be recaptured by giant plasma-membrane vesicle (GPMV) upon CC contact, further confirming its biophysical nature independent of intracellular signaling cascades. Our results, therefore, suggest a potential biophysical interaction between the CC and the plasma membrane, leading to an inflammatory milieu via sudden collapse of the latter as a consequence of cholesterol extraction.

## Materials and Methods

### Mice

Mice, all C57BL/6, were housed at Laboratory Animal Research Center of Tsinghua University. The wild type and *Il1r1^−^*^/^*^−^, Apoe^−/−^, Casp1^−^*^/^*^−^, Nfkb1^−^*^/^*^−^*mice were from Jackson Laboratory. *Casp11^−^*^/^*^−^, Casp11^tg^* were gifts from Dr. V. M. Dixit (Genetech, Inc.). *Nlrp3^−^*^/^*^−^* was a gift from Dr. Z. F. Jiang (Peking University). *Lyz2cre* was a gift from Dr. L. Wu (Tsinghua University). *Ripk3^−^*^/^*^−^Casp8^−^*^/^*^−^* was a gift from Dr. X. Lin (Tsinghua University). *GFP* transgenic was a gift from Dr. H. Qi (Tsinghua University). *Syk^fl/fl^* was generated by a commercial vender (Biocytogen). The gene-deficient mice were genotyped with the following primers: for *Casp1* were GAGACATATAAGGGAGAAGGG and ATGGCACACCACA GATATCGG and TGCTAAAGCGCATGCTCCAGACTG; for *Casp11* were TGAAATGCATGTACTGAGAGCAAGG and CAATTGACTTGGGGATTCTGG and GTCAGAGATGAAAGACTTTGCTGC; for *Nlrp3* were GCTATTCGGCTATGACTGGG and ACTTTCTCGGCAGGAGCAAG and TTGCCA CTGCTTATGTCCC and GCACACACACCTCCCTAACA; for *Nf*κ*b* were GCAAACCTGGGAATACTTCATGTGACTAAG and ATAGGCAAGGTCAGAATGCACCAGAAGTCC and AAATGTGTCAGTTTCATAGCCTGAAGAACG; for *Il1r* were CTCGTGCTTTACGGTATCGC and GGTGCAACTTCATAGAGAGATGA and TTCTGTGCATGCTGGAAAAC. All experiments performed were approved by the animal research committee of Tsinghua University.

### Cells

BMDM was produced as previously described (19). iBMDM (immortalized) cell line was a gift from Dr. X. Lin (Tsinghua University). CD4^+^ T cell was isolated with mouse CD4^+^ T cell isolation kit from Stemcell (19852A). 3T3 cell line was a gift from Dr. H. Qi (Tsinghua University). Mouse endothelial cells were isolated as described elsewhere (20). MDCK, RAW264.7, THP-1 cell line were from ATCC. BMDC and I.P. Mac was produced following the protocols in Current protocols in Immunology (2011). DC1940 cell line was a gift from Dr. L. Wu (Tsinghua University).

### Reagents

All reagents were purchased from Sigma-Aldrich unless otherwise indicated. Annexin V-FITC/PI Apoptosis Detection Kit was purchased from 4A Biotech (FXP018). LDH Cytotoxicity Assay Kit and propidium iodide were purchased from Beyotime (C0016, C1002 and ST512). DNA transfection reagent was purchased from Neofect (TF20121201). LPS transfer reagent was purchased from Promega (Fugene E231A). APC-Ly-6G ab was purchased from Sungene Biotech (M100L7-11A). FITC-Ly-6B.2 ab was purchased from GeneTex (GTX43417). Pronase was from Roche (18572720). CSFE and mouse TNFα were purchased from BioLegend (79898, 575202). Cycloheximide was purchased from Santa Cruz (sc-3508B). TRIzol reagent was purchased from Thermo (Ambion 15596018). Reverse transcribe kit and DNase I were purchased from TaKaRa (RR047A, 2212). RT-PCR super mix was purchased from Transgen biotech (AQ401). z-VAD-FMK, z-IETD-FMK, and z-LEHD-FMK were purchased from R&D system (FMK001, FMK007, and FMK008). Calpeptin was purchased from Enzo Life Sciences (BML-PI101). Cholesteryl BODIPY was purchased from Avanti Polar Lipid (810255P). Mouse IL-1β ELISA kit was purchased from eBioscience (88-7013). Membrane filter 0.22 µm SSWP was purchased from Millipore (SSWP02500). [1,2-^3^H(N)]-cholesterol was purchased from PerkinElmer (NET139250UC). Sulfo-NHS-LC-Biotin was purchased from APExBIO (A8003). Streptavidin was purchased from NEB (N7021S). MLKL and GSDMD shRNA were purchased from Sigma-Aldrich TRC shRNA libraries and TRC IDs are TRCN0000360818 (MLKL), TRCN0000219620 (GSDMD1), TRCN0000198776 (GSDMD2). SHC002 (non-target shRNA) was used as negative control.

### Crystals

MSU was made as previously described (21). Cholesterol crystal was made by cholesterol re-precipitation followed by ultra-sonication. Briefly, cholesterol powder was dissolved in warm acetone and then cooled down to precipitate cholesterol. The precipitated solids were washed with ddH_2_O three times, then dried off. PBS was added into the cholesterol crystal to reach a concentration for 25 mg/ml. The suspension was then treated with needle ultra-sonication to break large crystals into smaller ones (power: 30%, time: 60 min) to reach an average size around 1 µm. The sonication time was reduced to harvest larger crystal (10–20 μm: 5 min, 5–10 µm: 10 min, 2–5 µm: 30 min).

### Neutrophil Infiltration Assay

Supernatants from crystal-treated BMDM were collected by centrifugation followed with filtration through a 0.22-µm pore membrane. Crystals in saline or the supernatants were injected i.p. The peritoneal lavage fluid was collected 16 h after the injection. Briefly, mice were euthanized and its peritoneum was exposed. The peritoneum was lavaged with 10 ml ice-cold PBS containing 5 mM EDTA. Cells were stained with APC-Ly6G and FITC- Ly6B.2 following manufacturer’s instructions. Total cells number was counted with a hemocytometer then the samples were read with C6 Flow Cytometer. FACS data were analyzed with FlowJo V10.

### LDH Release Assay

Cell culture medium was replaced with Opti-MEM 2 h before stimulus treatment. Supernatants from the treated cells were collected and centrifuged (10,000 *g* 5 min) to remove solid particles. 20 µl each of lactate, INT, and diaphorase were combined to produce the reaction mixture. 120 µl of the supernatant from each sample was transferred to a new 96-well plate and mixed with 60 µl reaction mixture for 30 min at RT. Serum-free medium was used as the 0% control and lysate of the untreated cell was used as the 100% maximal release. Samples’ absorbance was read at 490 nm. LDH release from treated cells was determined based on a standard curve.

### Cell Death

Cells were collected after the indicated treatments and resuspended with Annexin V binding buffer which contains suggested concentration of Annexin V-FITC. 15 min later, supernatant was discarded by centrifugation. The cells were resuspended in the binding buffer with PI. Samples were read with C6 flow cytometer immediately. FACS data were analyzed with FlowJo V10.

For PI staining assay, cells were harvested and washed with PBS twice. CSFE were diluted in PBS and mix with cell suspension with a final concentration of 10 nM CFSE and 10^7^ cells/ml and then incubated for 15 min at RT. An equal volume of culture medium was added into the cells and wait for 5 min. Cells were washed and resuspended with culture media and plated in 24-well plates. After the treatment, supernatants were removed and PI (500 nM in PBS) was added into the well followed by a 2-min incubation. Fluorescent images were captured at 488 and 561 nm with fluorescent microscope. CFSE and PI-positive cell numbers were analyzed with CellProfiler 2.1.1 and project module MOD.cpproj. The proportion of PI-positive cells in total CSFE-positive cells was regarded as the cell death ratio.

### Atherosclerosis Model

*Apoe^−/−^* mice were crossed with *Casp1^−/−^, Casp11^−/−^, Nlrp3^−/−^, Il1r1^−/−^*, and *Nfkb1^−/−^*. Mice were fed with high-fat diet (21% fat, 0.15% cholesterol, MD12015, Medicience, Ltd.) or the control diet starting 3 weeks of age for 12 weeks. Each group has almost equal male and female mice. Mice were then sacrificed and the aorta was isolated. Heart perfusion was performed to prevent clotting. The connective tissue and fat around the aorta were removed with tweezers under dissecting microscope carefully. 3% (w/v) of Oil Red O in 2-protanol was used as the stock staining solution (22). Oil Red O working solution was diluted from the stock with ddH_2_O at 6:4 ratio. Stained samples were rinsed with 70% 2-propanal quickly then moved to Oil Red O working solution and stain for 30 min on a slow shaker. The samples were rinsed again in 70% 2-propanal for 10 s and returned to ddH_2_O. The aortas were opened en face by tweezers and ophthalmic scissors with acupuncture needles fixing the aorta. The aortas were imaged with microscope and the area of red plaques was determined with microscope (Olympus) image program which based on the pixel size.

### shRNA Knock Down Assay

Plasmids including shRNA and the package construct (Δ8.9 and VSVG) were purified from transformed *E. coli* with EndoFree Plasmid Midi Kit (CWBIO, CW2105S). 293FT cells were cultured in a 25 cm^2^ flask with 60–80% confluency. Culture media were replaced 2 h before DNA transfer. 2 µg shRNA plasmid, 1 µg Δ8.9, and 1 µg VSVG were mixed in 50 µl of OptiMem medium. 4 µl DNA transfection reagent was added to the mixture OptiMem medium and incubated at RT for 20 min. The mixture was added into 293FT medium carefully. 6 h later, the media were replaced with DMEM containing 20% serum. Virus-containing supernatants were harvested 60 h after the DNA transfection and then centrifuged at 1,000 *g* for 10 min to discard the debris. iBMDM cells were cultured in 24-well plates with 20–30% of the area covered by cells. Polybrene (final concentration 8 µg/ml) was mixed with the virus-containing supernatant and the mixture was transferred into the cell culture. The plates were centrifuged at 500 *g* at 30°C for 90 min and then exchanged into regular culture medium. 48 h after the virus infection, 2 µg/ml puromycin was added to the medium. Puromycin-resistant cells were harvested several days later and their RNA was extracted with TRIzol Reagent following manufacturer’s instructions. RNA concentration was determined by Nanodrop 2000 (Thermo). 1 µg RNA was reverse transcribed to cDNA immediately with a reverse transcription kit (Takara, RR047A). Gene expression was determined by real time PCR.

### RT-PCR

All procedures were performed per manufacturer’s instructions of the SYBR Green qPCR SuperMix (Transgen Biotch, AQ101). Primers for mouse MLKL were AATTGTACTCTGGGAAATTGCCA and TCTCCAAGATTCCGTCCACAG. Primers for mouse GSDMD were TACTGCCTTCTGAACAGGAA and GTCACCACAAACAGGTCATC. β-actin expression was used as internal control and primer sequences were TTGCTGACAGGATGCAGAAG and ACATCTGCTGGAAGGTGGAC.

### LPS Intracellular Delivery

For LPS delivery, cells were cultured on 24-well plate, and the media were replaced with 300 µl Opti-MEM. LPS (2 µg/ml final) and transfect reagent Fugene (0.25% v/v final) were mixed in 50 µl Opti-MEM and incubated at RT for 15 min before being slowly added into the cell culture.

### Atomic Force Microscopy

All crystals were glued to cantilever (Arrow TL- 1, Nanoworld) with epoxy (21) at least 12 h before use. All attraction forces between cell and crystal were measured with JPK CellHesion (JPK) (23). Briefly, the measurement was carried in the relative force feedback contact/tapping mode (IP gain: 50 Hz; IG gain: 0.001 Hz; correct baseline: 1; relative set point: 0.5 nN; *z* length: 50 µm; extend delay: 0 s; constant height). Data were analyzed with JPK Data Processing. Maximum binding forces were calculated and plotted. Each dot presents a single measurement.

Attraction forces between crystal and lipid monolayer were measured with JPK NanoWizard II (JPK). All set up was the same as the JPK CellHesion except *z* length was 10 µm. AFM scanning mode was used to confirm domain formation.

### Cholesterol Transfer Assay

10 µg of BODIPY-cholesterol in DMSO (20 mg/ml) was mixed with 2 mg of MβCD in PBS (100 mg/ml). Bath sonication at 37°C was used to solubilize the cholesterol. BODIPY-cholesterol and MβCD mix solution was added to cells in serum-free culture medium (10% v/v) and incubated at 37°C for 20 min. Cells were washed with PBS three times and replaced with fresh culture medium. They were imaged with fluorescent microscope to confirm the labeling efficiency. The cells were returned to culture to allow cells to settle on the bottom. 200 µg/ml CC, MSU, or Silica crystals were added to the cells and incubated for different durations, and eventually lysed with 1% NP40. The lysates were centrifuged at 500 *g* for 5 min. The supernatants were discarded and the residual crystals were washed with 1% NP40 two more times. The washed crystals were resuspended with ddH_2_O and the fluorescence associated with the crystals was analyzed with C6 flow cytometer or fluorescent microscope.

Cells were labbeled with NHS-LC-biotin (Thermo, 21327) first following manufacturer’s instruction. Then Labeling cell surface with [1,2-^3^H(N)]-cholesterol followed the same procedure described above except cholesterol-[^3^H] was diluted in cholesterol and MβCD mixture (1:100 molar ratio in PBS). Cholesterol-[^3^H] labeled cells were treated with crystals for 24 h and then lysed with 10% NP40 for 1 min. Cell lysates and crystals were centrifuged at 500 *g* for 5 min. The crystals were washed with 1% NP40 then resuspended with PBS. The crystals in the suspension were mixed with streptavidin conjugated agarose beads (Thermo, 20347) for 15 min to remove cell membrane debris. Collect the crystal with spin column (Thermo, 69725) then mix the crystals with ULTIMA GOLD (PerkinElmer, 6013327) and read with the liquid scintillation counter.

### GPMV Membrane Integrity Assay

BMDM of *GFP*^tg^ mice was cultured as previously described (24). The cells were washed with ice-cold PBS (pH 8.0) three times and cell numbers were counted. They were resuspended at a concentration of 3 × 10^7^ cells/ml in PBS (pH 8.0). 1 mg of Sulfo-NHS-LC-Biotin was added into the suspension for 1 ml of the preparation and then incubated at RT for 30 min. Biotin was quenched by washing the cell with PBS + 100 mM glycine three times. The cells were plated in the 6-well plate with 3 × 10^6^ cells per well and the GPMV was produced following a published protocol (25): The GPMV-containing supernatant was collected and moved to a round glass slide which had been coated with Streptavidin for 2 h at 37°C. 2 h later, the supernatant was discarded and GPMV attached to the glass slide was washed with GPMV buffer twice. GPMV attachment was confirmed by microscope. A cantilever which had been glued with a crystal on the top was first moved to settle on the GPMV using the relative force feedback contact/tapping mode (IP gain: 50 Hz; IG gain: 0.001 Hz; correct baseline: 1; relative set point: 1 nN; *z* length: 5 µm; extended delay: 10,000 s; retract delay: 0 s; constant height). The fluorescence of GFP inside GPMV was imaged every 20 or 30 s for 2 h. The image data were analyzed in ImageJ. Briefly, mean fluorescence intensity of GPMV was measured and the value of Intensity _GPMV_/Intensity _Background_ was calculated. This *I*_G_/*I*_B_ was normalized to its initial value to eliminate fluorescent intensity difference between individual GPMV. In order to digitalize the fluorescence change of GPMV, we subtracted *I*_G_/*I*_B_ value of any given point by the same *I*_G_/*I*_B_ of the same vesicle after 5 min. The maximal differential from each GPMV was used to plot Max MFI reduction (*K*_5min_ = normalized *I*_G_/*I*_B 5min before_ − normalized *I*_G_/*I*_B after_).

### Lipid Bilayer Preparation

Lipid bilayers were prepared according to a published protocol (26). Briefly, DOPC, sphingomyelin, and cholesterol were mixed and dissolved in chloroform solution with 1 mg total lipid/50 μl at the indicated ratios. Chloroform was dried off in vacuum for 2 h. The remaining lipids were resuspended with 100 µl PBS. 10 µl of the lipid suspension was added to 150 µl of supported lipid bilayer buffer (SLB, 150 mM NaCl, 10 mM HEPES, pH 7.4) and sonicated in a bath sonicator to yield small unilamellar vesicles. A clean mica surface was obtained by peeling off the outer layer with adhesive tape. The lipid mixture was placed on the mica and CaCl_2_ (final concentration 3 mM) was added to the mixture. The preparation was incubated at 37°C for 2 min then at 65°C for 15 min. The bilayer was washed with 65°C SLB 15 times then cooled slowly.

### Statistics

All plot graphs show means with SEM. Statistical analysis for each independent experiment was performed with an unpaired, Student’s *t*-test. A *p* value of less than 0.05 was considered significant. *<0.05; **<0.01; ***<0.001; N.S., not significant. All experiments were repeated at least three times independently except Figures 2E,F where pooled data from several experiments were used.

## Results

**Figure 1:**
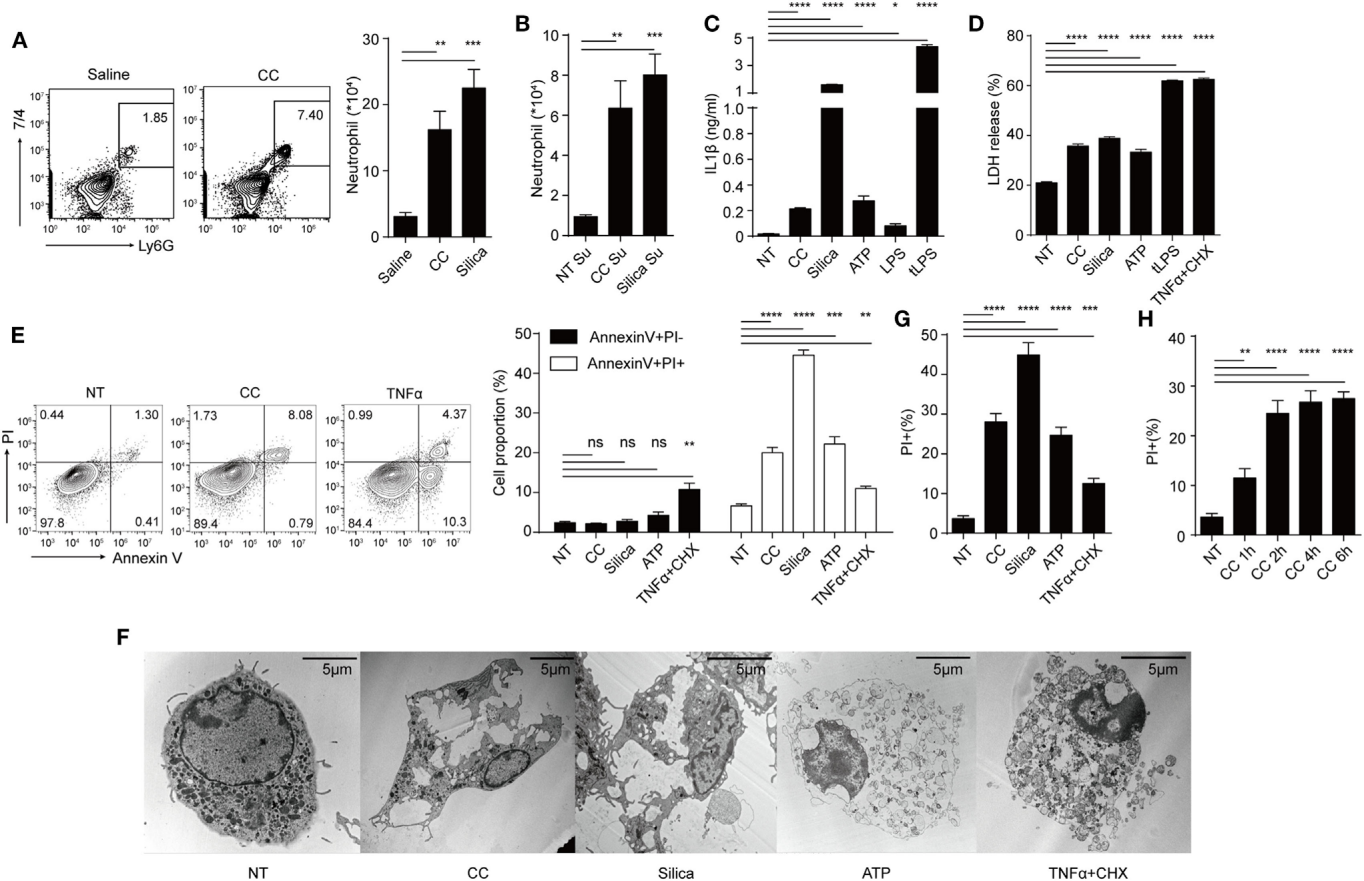
Cholesterol crystals (CC) induce both NLRP3 inflammasome activation and a rapid necrotic cell death in the phagocytic cells. **(A)** C57BL/6 mice were i.p. injected with 500 µg of Silica or CC in 1 ml saline and rested for 16 h. Intraperitoneal cells were harvested and stained with Ly6G-APC and 7/4-FITC. The right panel is the bar graph of FACS plots on the left. *n* = 4 (*n* indicates the number of repeats of each group), *N* = 3 (*N* indicates the number of biological repeats of the experiment). Two-tailed Student’s *t* analysis results for all figures are indicated as follows: *p* < 0.001: ***; *p* < 0.01: **; *p* < 0.05: *; *p* at 0.05 or larger was considered non-significant: ns. **(B)** Supernatants of treated BMDM (CC: 1 mg/ml, Silica:500 μg/ml for 8 h) in 12-well plates (about 4 × 10^5^ cells) were collected and injected i.p. 16 h later intraperitoneal cells were harvested and stained as in **(A)**. *n* = 4. *N* = 3. **(C)** BMDM were primed with Pam3csk4 (100 ng/ml) for 12 h and then treated with indicated stimulus for 12 h (CC:500 μg/ml, Silica: 100 µg/ml, ATP:5 mM, LPS: 2 µg/ml, tLPS: LPS was packed into liposome with final concentration 2 µg/ml as described in the Section Materials and Methods). IL-1β concentration was determined by ELISA. *n* = 6. *N* = 3. **(D)** BMDM were primed then treated as in **(C)** for 12 h and the cell death was determined by LDH assay. TNFα + CHX:100 ng/ml TNFα plus 50 µg/ml Cycloheximide. *n* = 4. *N* = 3. **(E)** BMDM were primed then treated as in **(D)** except treatment duration (CC: 2 h, Silica: 1 h, ATP: 2 h, TNFα + CHX: 6 h) then were collected and stained with Annexin V-FITC and PI. The right panel is the bar graphs of FACS plots on the left. *n* = 4. *N* = 4. **(F)** TEM picture of BMDM primed then treated as in **(E)**. **(G)** BMDM were primed then treated as in **(E)** except stained with PI. This is to indicate that using PI + staining alone faithfully recaptures nearly cell death events as in **(E)**. *n* = 6. *N* = 3. **(H)** BMDM were primed then treated with CC (500 µg/ml) for different durations then stained with PI. PI + percentages are shown. *n* = 6. *N* = 3.

We first studied the activation parameters of macrophages stimulated by CC. Figure 1A shows i.p. injection of CC induced the infiltration of neutrophils after 16 h, similar to silica crystals (24). To understand the factors promoting such an *in vivo* response, macrophages were treated with CC and the culture supernatants were similarly injected i.p. Figure 1B shows that the supernatants from both silica and CC-treated cells also induced this inflammatory response. As cytokine production and necrosis can both release pro-inflammatory agents (27), IL-1β, IL-1α, IL-6, and TNFα production and cell death were analyzed. IL-1β was readily detected in the supernatant of BMDM/CC coculture, suggesting that NLRP3 inflammasome was activated as reported previously (Figure 1C) (5). CC also induced other pro-inflammatory agents (Figure S1A in Supplementary Material) indicating a broad spectrum of inflammatory response to this crystal. Simultaneously, CC triggered substantial cell death, as determined by LDH release and Annexin V PI staining (Figures 1D,E). In contrast to TNFα, CC-treated cells displayed typical necrotic plasma-membrane rupture (Figure 1F) and did not show an intermediate stage of Annexin V^+^ PI*^−^* that is associated with apoptosis (Figure 1E). Therefore, CC-mediated cell death was nearly captured in full by PI-positive staining alone (Figures 1E,G). This cell death was detectable within 1 h (Figure 1H), again suggesting an apoptosis-independent cell death programing. Considering the high spontaneous LDH release (NT in Figure 1D) and LDH release requires larger pore on cell membrane, which may miss the initial loss of membrane integrity, than Pi staining. We decided to use Pi staining assay as the main readout for cell death. As atherosclerosis progresses, macrophages, DCs, smooth muscle cells, and T cells are found inside the lesion intima (1). We tested CC cytotoxicity on those cells (Figures S1B,C in Supplementary Material). CD4^+^ T cells, fibroblast (3T3), and endothelial cells (MuEC and MDCK) showed little necrotic cell death in CC treatment while DCs and macrophages exhibited Annexin V^+^ PI^+^ staining. We also tested cytotoxicity mediated by CC of different sizes. Figure S1D in Supplementary Material shows that cell death was more prominent with CC less than 5 µm. Therefore, CC can induce both NLRP3 inflammasome activation and a rapid necrotic cell death in the phagocytic cells.

**Figure 2:**
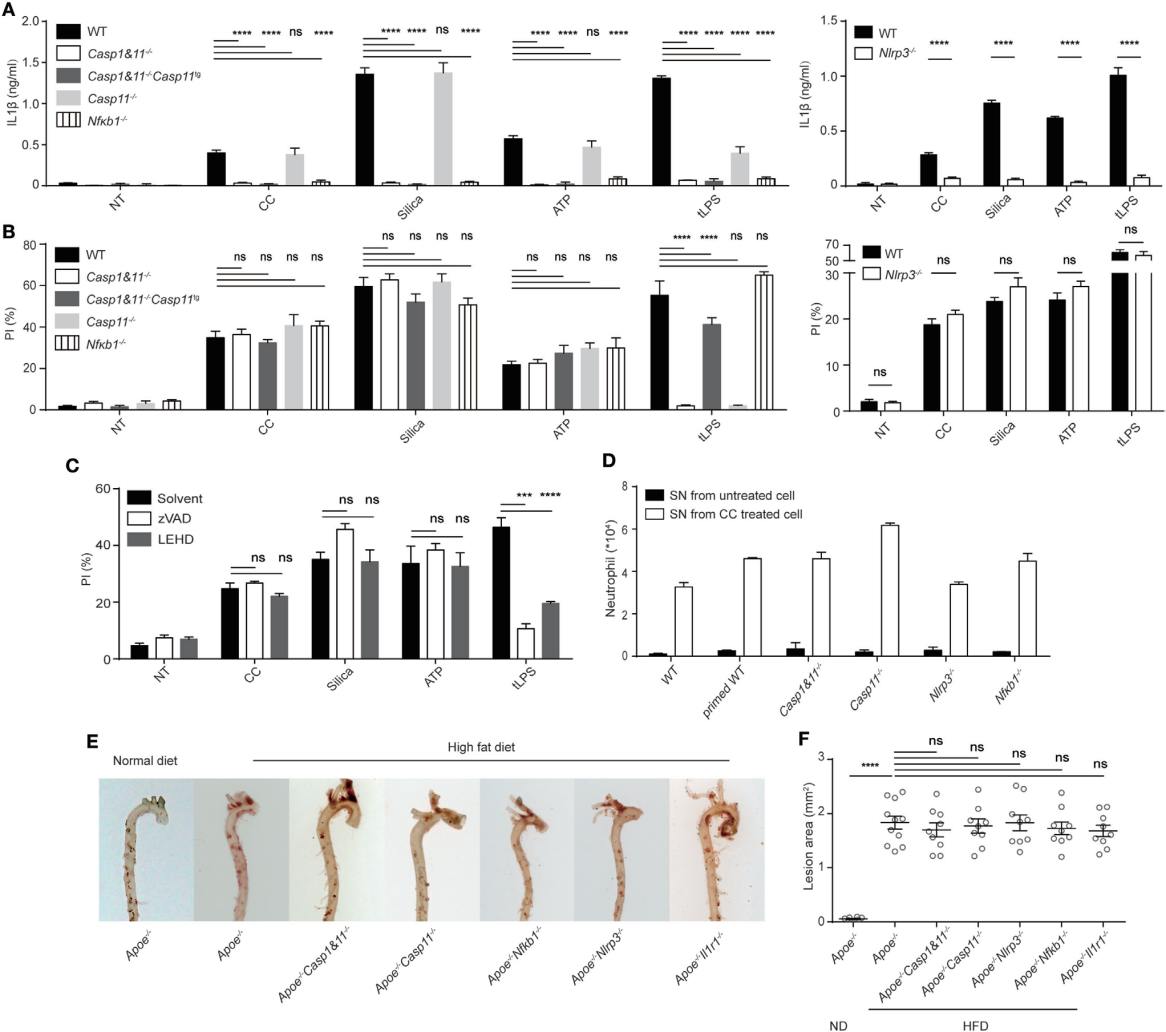
Cholesterol crystals (CC)-mediated cell death and its pro-inflammatory property is unrelated to NLRP3 inflammasome. **(A)** BMDM with indicated genotypes were primed with Pam3csk4 then treated with indicated stimuli for 6 h as in Figure 1C. IL-1β was determined by ELISA. *n* = 4. *N* = 4. **(B)** BMDM were primed and then treated as in Figure 1C except treatment duration (CC: 2 h, Silica: 1 h, ATP: 2 h, tLPS: 6 h). Cell death was determined by PI staining. *n* = 6. *N* = 5. **(C)** BMDM were primed then pretreated with Caspase inhibitor (z-VAD-FMK: 10 µM, z-LEHD-FMK: 2 µM) 0.5 h before the stimulus treatments as in **(B)**. Cell death was determined as in **(B)**. *n* = 6. *N* = 5. **(D)** BMDM from WT or gene-deficient mice were cultured. Supernatants were collected and injected identical to Figure 1B. 16 h later, intraperitoneal cells were harvested and stained as in Figure 1B. *n* = 3. *N* = 3. **(E)** Inflammasome component-deficient mice were crossed to *Apoe^−/−^*. Homozygous mice were fed with high-fat diet for 12 weeks and then the aorta from these mice were stained with Oil Red O. **(F)** The aortas in E were opened *en face* (Figure S2 in Supplementary Material) and imaged with microscope. The area of red plaques was determined with Olympus microscope image program based on pixel size.

As Caspase 1/11 activation leads to pyroptosis, a form of cell death resulting from plasma-membrane permeability change (16, 28), the role of NLRP3 inflammasome in CC-mediated cell death was analyzed. Primed, gene-deficient BMDM were treated with CC, silica, ATP and liposome-delivered LPS (confirmation of gene deletion is shown in Figure S2A in Supplementary Material). NLRP3-deficient BMDM failed to produce IL-1β as expected (Figure 2A). NFκb and Caspase 1 were both critically involved in IL-1β production, which are known to regulate the priming and the production phases of IL-1β, respectively (29) (Figure 2A). Interestingly, Caspase1 deficiency did not impact CC-mediated cell death. The killing efficiency was not altered in *Casp1&11^−/−^* or the Caspase 11 transgene version of *Casp1&11^−/−^* BMDM (Figure 2B) (30). LDH released to the medium confirmed the result in Pi staining assay (Figure S2D in Supplementary Material). In line with this finding, *Nlrp3^−/−^* macrophages showed a degree of necrotic cell death similar to WT cells (Figure 2B). As well-known activators of NLRP3 inflammasome, it seems to be surprising that ATP and silica-induced cell death does not rely on NLRP3 inflammasome, However, there have been reports suggesting that ATP, Al(OH)_3_, Silica, CPPD, LLOMe-induced macrophage cell death proceeded unimpeded under *Nlrp3* or *Casp1* deficient background (31–34). This is likely that those triggers may mediate some form of membrane damage or cellular stress independent of NLPR3/Caspase 1 pathway. Pan caspase inhibitor zVAD and Caspase 9 inhibitor LEHD again did not alter the rate of cell death (Figure 2C). Therefore, CC-mediated cell death is different from pyroptosis, and is unrelated to the roles of Caspase 1 and NLRP3 in IL-1β production.

As CC promote a pro-inflammatory milieu in Figures 1A,B, we compared necrotic death-associated inflammation to that mediated by simultaneous IL-1β production. While the CC-treated BMDM released stimulatory factors in supernatants that promoted peritoneal neutrophil recruitment, this effect was minimally altered in *Casp1&11^−/−^, Nlrp3^−/−^*, or *Nfkb1^−/−^* BMDM (Figure 2D), suggesting that the inflammatory IL-1β production was not the sole factor promoting inflammatory responses in our setting, at least in the light of the necrotic death-mediated releases of intracellular contents. *Apoe^−/−^* (a component of LDL transport system) mice have increased cholesterol levels and heightened plaque formation when fed with a high-fat diet (35). In these mice, deficiencies in Caspase1 and 11, NLRP3, IL-1 receptor, and NFκb did not alter the arteriosclerotic plaque formation (Figures 2E,F; Figure S2B in Supplementary Material). This result disagrees with a previous report that NLRP3 activation was involved in atherosclerosis development (5), instead in line with later reports suggesting the lack of influence by this inflammasome complex (11, 12). While these discrepancies could be results of experimental settings, our data suggest the possibility that necrotic cell death may function as a pro-inflammatory signaling in atherosclerotic progression independent of the NLRP3 inflammasome activation. Furthermore, we tested which kind of cytosolic contents is inflammatory. CC-treated cell supernatant was treated with DNAase, pronase (Figure S2C in Supplementary Material). The result shows that both proteolytic and DNA digestions reduced neutrophil infiltration, suggesting that necrotic releases of nucleic acids and proteins are indeed inflammatory (36, 37).

**Figure 3:**
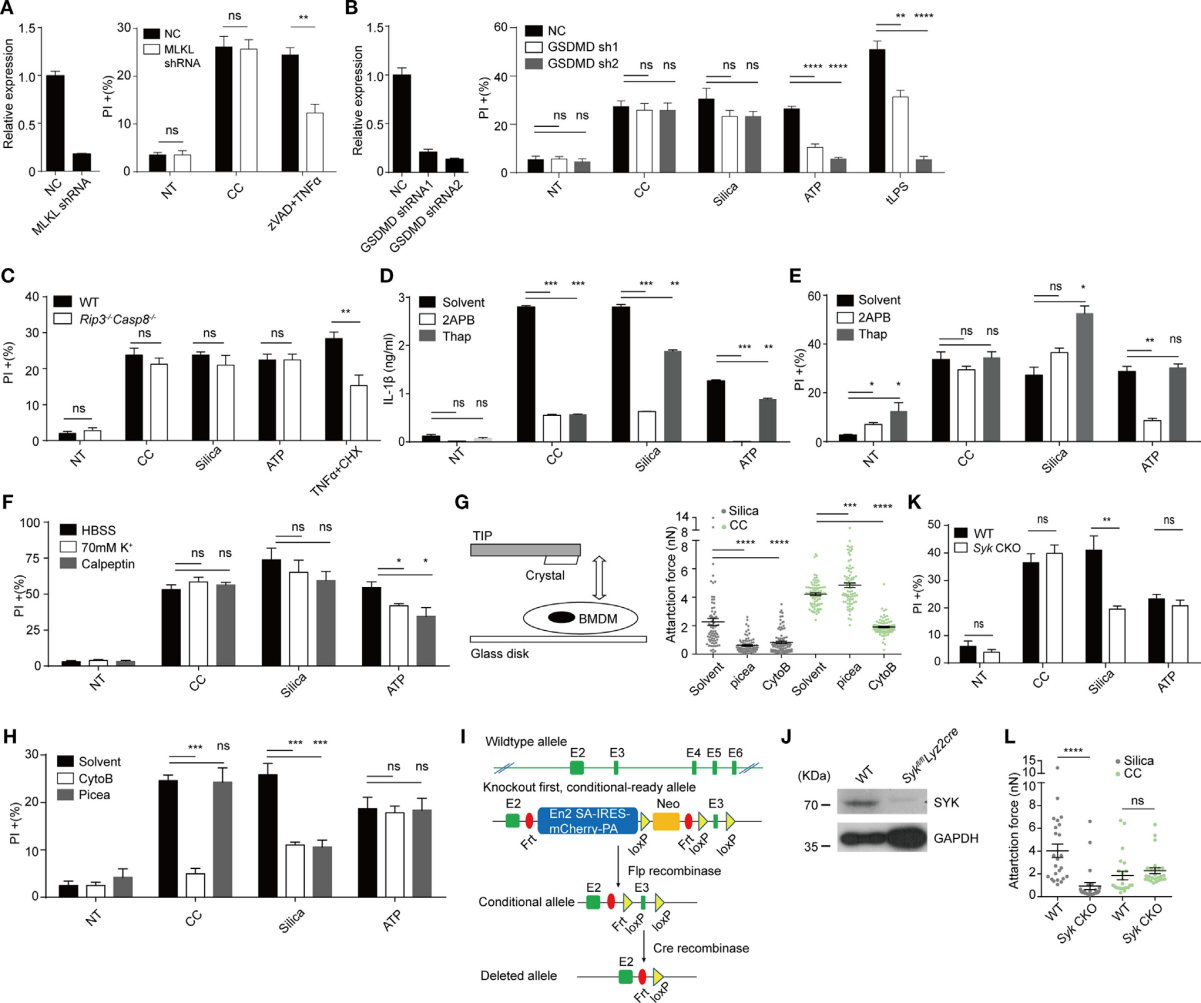
Cholesterol crystals (CC)-mediated cell death is unrelated to mixed lineage kinase domain-like kinase (MLKL), gasdermin D (GSDMD), CASP8, Ca^2+^ influx, K^+^ efflux and SYK. **(A)** Left: MLKL was knocked down in iBMDM by shRNA. Gene expression was determined by Q-PCR. Right: iBMDM were treated with CC for 2 h or zVAD (10 µM) plus TNFα (50 ng/ml) and CHX (50 µg/ml) for 12 h and then the cell death was determined with PI staining. *n* = 6. *N* = 5. **(B)** Left: GSDMD was knocked down in iBMDM by shRNA as in **(A)**. Right: iBMDM were primed then treated identical to Figure 1C except for the treatment duration (CC: 2 h, Silica: 1 h, ATP: 2 h, tLPS: 6 h). Cell death was determined with PI staining. *n* = 6. *N* = 4. **(C)** BMDM with Caspase 8 deficiency were treated and stained as in Figure 1G. *n* = 6. *N* = 3. **(D)** BMDM were primed then pretreated with 50 µM 2APB or 1 µM Thapsigargin for 0.5 h before stimulus treatment as in Figure 1C. 6 h later, IL1β production was determined by ELISA. *n* = 4. *N* = 3. **(E)** Identical to **(D)** except for the treatment duration (CC: 2 h, Silica: 1 h, ATP: 2 h). Cell death was determined with PI staining. *n* = 5. *N* = 6. **(F)** BMDM were primed and the media were exchanged into HBSS, 70 mM K^+^ HBSS, or HBSS containing 100 µM Calpeptin 15 min before stimulus treatment as in Figure 1C except for the treatment duration (CC: 2 h, Silica: 1 h, ATP: 2 h). Cell death was determined with PI staining. *n* = 6. *N* = 5. **(G)** The left panel shows the schematic of how attraction force between crystals and cells was measured by AFM. Right panel: BMDM were primed and pretreated with piceatannol (50 µM) or Cytochalasin B (1 µg/ml) for 20 min. The attraction force after 5 s contact time was measured by AFM. *N* = 3. **(H)** BMDM were pretreated as in **(G)** before being treated with stimuli as in Figure 1C except for the treatment duration (CC: 2 h, Silica: 1 h, ATP: 2 h). Cell death was determined by PI staining. *n* = 6. *N* = 3. **(I)** The design of Syk CKO. **(J)** Syk expression in *Syk* CKO BMDM was examined by western blot. *N* = 3. **(K)** *Syk* CKO and WT BMDM were primed and treated with stimuli as in Figure 1C except for the treatment duration (CC: 2 h, Silica: 1 h, ATP: 2 h). Cell death was determined by PI staining. *n* = 6. *N* = 7. **(L)** The attraction forces after 5 s contact time between *Syk* CKO BMDM and crystals were measured by AFM. *N* = 3.

We wondered if CC-mediated cell death was regulated by any known cell death pathways independent of Caspase 1&11 and NLRP3 (38). In recent years, two membrane-targeting, pyroptosis-inducing mechanisms have been proposed. MLKL, a component of necroptosome, mediates necroptosis typically involving a death receptor (i.e., TNFR1), RIPK1 and RIPK3. MLKL is phosphorylated by RIPK3 and mediates the plasma-membrane disruption. This pathway is particularly active in the absence of Caspase activation (39, 40). Caspases 1 and 11 on the other hand process GSDMD, promoting its N-terminal fragment to translocate to the plasma membrane to form a pore-like structure, leading to pyroptosis (16). We tested the involvement of MLKL and GSDMD by gene knockdown. MLKL knockdown macrophages (Figure 3A) showed cell death similar to the control-treated cells, a result collaborated by the lack of cell death inhibition by necrostatin-1 (data not shown), an inhibitor of RIPK1. In comparison, TNFα + ZVAD-induced cell death was reduced (Figure 3A) (41). GSDMD knockdown also failed to reduce CC-induced, although blocked tLPS-induced cell death (Figure 3B). We tested potential involvement of TNFR1-Caspase 8 axis as it is known to regulate apoptosis and alternative Caspase 1 activation (42). *Rip3^−/−^Casp8^−/−^* did not show any change of CC-mediated cell death (Figure 3C). The uptake of solid structures induces Ca^2+^ signaling; Ca^2+^ influx can lead to mitochondria ROS production that triggers executioner caspases. In addition, massive Ca^2+^ increase can activate the calpain system that results in endo-reticulum structural damage (17, 43). Figures 3D,E show that while IL-1β production was sensitive to 2APB (IP3 receptor blocker) and Thapsigargin (Sarco/endoplasmic reticulum Ca^2+^ ATPase inhibitor), neither treatment resulted in reduced cell death. A general calpain inhibitor, calpeptin (44), and high extracellular K^+^ also failed to inhibit the cell death (Figure 3F; Figure S2E in Supplementary Material). Therefore, our results suggest that CC appeared to mediate a necrotic cell death without involving the common pathways such as apoptosis, pyroptosis, ROS production, and calpain-based self-internal digestion.

To study the nature of CC-mediated cell death, atomic force microscopy (45) -based single cell force spectroscopy (SCFS) was used to measure its binding affinity to the cellular membrane (23). Similar to silica crystal, CC induced a substantial binding force to macrophage cell surface and the affinity increased over time under a set contact force of 0.5 nN (data not shown). A typical Fc receptor-mediated phagocytosis of a solid particle is a complex process that requires an early phase cortical cytoskeleton remodeling regulated by GTPases such as CDC42, Rac1, and Rho (46) followed by signaling chains involving src family kinase, Syk as well as PI3Ks (46). Upon a particle binding, local actomyosin system generates the initial membrane contour that fully engages the solid target. This creates the initial binding affinity. Simultaneously, src family kinases phosphorylate ITAM motifs contained in phagocytic receptor complexes, which attracts Syk to the membrane. Syk then activates PI3K/PLC and other downstream signals for a full spectrum phagocytic activation (46). From a biophysical angle, the second stage, critically dependent on Syk, is characterized by irreversible strong binding forces (21, 23). In addition, Syk signaling also contributes to the restriction of receptor lateral diffusion, optimizing the signaling feedback loop during phagocytosis (47). The CC contact force was sensitive to actin polymerization inhibitor cytochalasin B, similar to Silica (Figure 3G). To our surprise, this force was completely intact in the presence of Syk inhibitor piceatannol (Figure 3G). In contrast to cytochalasin B, piceatannol had minimal impact on CC-mediated cell death (Figure 3H).

In our lab, solid particle phagocytosis (including MSU, basic calcium phosphate, calcium pyrophosphate dihydrate, allopurinol, silica, prefabricated latex beads, and several preparations of alum salt) has always shown a complete dependence on Syk, exhibiting a strong binding force as determined by AFM (data not shown). CC has been the only exception. This result suggested that strong affinity measured by SCFS was not mediated by typical phagocytosis signaling. Considering the off target/unintended effects of inhibitors and the lethality of the full Syk deficiency (vascular development defects) (48), we generated a conditional inducible deletion strain (Figure 3I). In brief, a Frt-framed box was inserted in between exons 2 and 3, which contained an En2-SA-IRES-mCherry-PA fragment and a neomycin cassette, with a loxP site in between. The exon 3 was additionally framed by a loxP pair. The original design led to a Syk-null genotype. A crossing to a full body Frt converted the original deficient strain into a conditional knockout allele. A further crossing to *Lyz2cre* deleted exon 3 to produce a strain with a restricted myeloid Syk deficiency. In comparison with the systemic deletion, these mice show no visible abnormality yet macrophages in this strain were largely devoid of Syk (Figure 3J). The cell death mediated by CC was unaffected by the Syk deletion, in sharp contrast to silica crystals (Figure 3K). The SCFS using Syk-deficient macrophages confirmed that the absence of Syk did not impact the binding, while the affinity of silica crystal to the macrophage was significantly reduced (Figure 3L). Therefore, the strong binding of CC to the plasma membrane does not rely on Syk-dependent phagocytosis.

**Figure 4:**
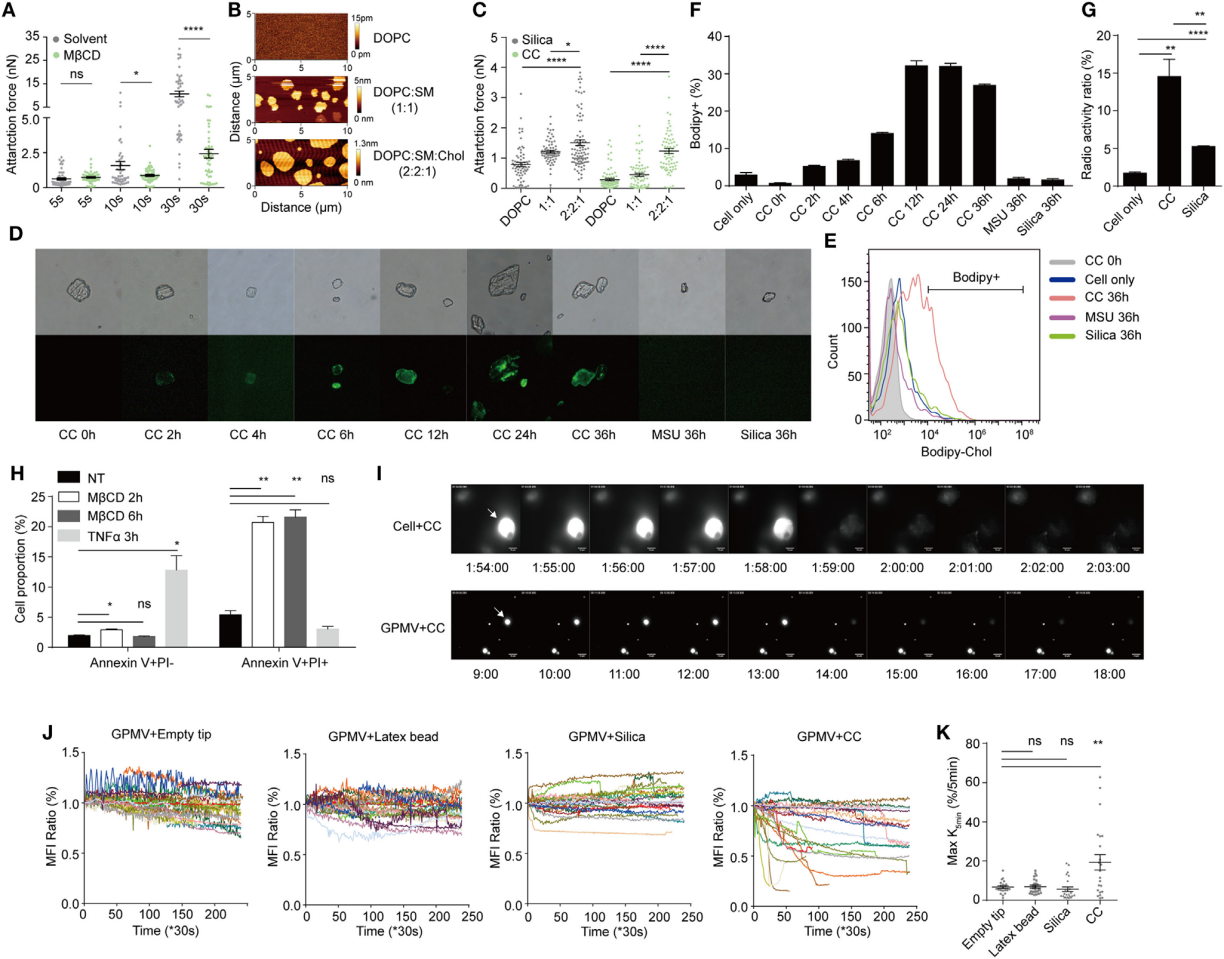
Cholesterol crystals (CC) extract cholesterol from membrane thus induce signaling free membrane rupture. **(A)** BMDM membrane cholesterol was depleted by MβCD (4 mg/ml) incubation in serum-free medium for 0.5 h. The attraction force between CC and cells after different contact times was measured by AFM. *N* = 3. **(B)** Lipid bilayers with or without cholesterol/SM were produced. The surface of the bilayer was scanned by JPK NanoWizard. The shade of color indicates the height of the surface. **(C)** The attraction forces between crystal and lipid bilayers were measured by AFM with a contact time of 30 s. *N* = 3. **(D)** BMDM were labeled with Bodipy-cholesterol delivered by MβCD and treated with CC (100 µg/ml), MSU (100 µg/ml), or silica (80 µg/ml). After different lengths of incubation at 37°C, cells were lysed and the fluorescence on collected crystals were observed with fluorescence microscope. **(E)** The collected crystals in **(D)** were analyzed with FACS. **(F)** The bar graphs of Bodipy-positive crystals in **(E)**. *n* = 3. *N* = 6. **(G)** BMDM was labeled with cholesterol-[H^3^] and treated with CC (100 µg/ml), silica (100 µg/ml). 24 h later, cells were lysed and the radioactivity of the collected crystals was detected. *n* = 4. *N* = 3. **(H)** BMDM were treated with MβCD (8 mg/ml) for 2 or 6 h or with TNFα plus CHX for 3 h. Cells were collected and stained with Annexin V and PI. Samples were analyzed by FACS. *n* = 4. *N* = 4. **(I)** Upper: A GFP-expressing BMDM was contacted by a CC constantly. GFP fluorescence of the cell was imaged every 20 s with a 488-nm laser in FITC channel. The pictures show the cell fluorescence around the moment of “explosion.” Numbers below the pictures indicate the time since crystal contact. Lower: a GFP-containing giant plasma-membrane vesicle (GPMV) was contacted by a CC constantly. The rest was identical to the upper panel. The white arrow indicates the crystal-contacted cell or GPMV. **(J)** Mean fluorescence intensity of GPMV and background was measured with ImageJ. Fold change (compared with initial value) curve of MFI_GPMV_/MFI_Background_ during CC and other crystal contact was calculated. Each curve represents a crystal-contacted vesicle. **(K)** Maximum reduction of normalized MFI_GPMV_/MFI_Background_ within 5 min during the contact was manually identified and plotted.

Our previous work on MSU crystal revealed that in the receptor-independent phagocytosis, MSU crystals preferentially engage membrane cholesterol. This binding sorts cell-surface lipids, particularly lipid rafts, and a spontaneous Syk activation (21). We wondered if cell membrane cholesterol was similarly involved in the CC binding. Upon depletion of membrane cholesterol by MβCD, the CC-mediated binding was significantly reduced (Figure 4A). We created a synthetic bilayer membrane with base DOPC, or in mixture with sphingomyelin/cholesterol (21). The quality of the membrane on a mica surface was confirmed with the scanning mode AFM (Figure 4B) which revealed well-defined lipid domains (49). The contact of cholesterol-containing bilayer generated higher binding force than DOPC alone (Figure 4C). Similar to other crystal growth, CC can expand in the presence of free cholesterol in the environment (50). We wondered if cell-surface cholesterol content was disturbed upon CC contact. BMDM were loaded with Bodipy-cholesterol and incubated with label-free CC. Interestingly, CC became Bodipy-positive after the co-incubation (Figure 4D). To verify that this transfer was contact-dependent, the cells and CC were separated in a transwell setup, and no Bodipy transfer was detected (Figures S3A,B in Supplementary Material). A detailed time course comparison showed that over time the labeled cholesterol from BMDM was gradually transferred to a percentage of CC, while this transfer was minimal for MSU and silica crystals (Figures 4E,F). MFI of CC as a whole was also higher than MSU and silica controls (Figure S3C in Supplementary Material). To exclude the possibility that this transfer was a result of Bodipy-cholesterol falling off the cell membrane, as Bodipy may change cholesterol hydrophobic property, tritium (^3^H)-replaced cholesterol was used to label BMDM and the cells were incubated with silica and CC. The crystals were then harvested and read with a scintillation counter. Figure 4G shows that CC preferentially attracted cholesterol while the radioactivity on silica was much less. These results demonstrate that CC can attract cell-surface cholesterol for their own growth, reminiscent of typical crystal growth that absorbs its precursor from the environment.

The scenario presented above implicates a possibility that depletion of membrane cholesterol mediates necrotic membrane rupture. To capture this event, we treated cells with MβCD. This treatment led to a necrotic cell death in contrast with the apoptosis induced by TNFα (Figure 4H). A CC was delivered by SCFS to be in contact with a GFP-expressing BMDM, and the setup was monitored by a microscope over time. Initially, the GFP signal remained steady under CC contact. However, the signal was suddenly lost. The cell in contact exhibited an “explosive” perfusion of GFP label (Figure 4I; Movie S1 in Supplementary Material). To confirm that such a death event was independent of cellular signaling, we generated GPMV, a “ghost” bubble exocytosed by phagocytes treated with PFA (25). GFP-expressing BMDM were pre-labeled with biotin, and the cells were treated with PFA to obtain GPMV. These GPMV were attached to glass surface coated with streptavidin for anchoring against free motion. CC glued to AFM cantilever was used to contact with these vesicles and the fluorescence intensity was again recorded by time-lapse videos (Figure 4I; Movie S2 in Supplementary Material). In comparison with silica crystals and latex beads, CC-contacted GPMV also displayed a higher frequency of “explosive” sudden loss of label, suggesting a catastrophic membrane rupture event (Figure 4J; Movies S2–S5 in Supplementary Material). In comparison, while GFP signal might wane with latex beads and silica, the loss was not significantly over the spontaneous loss in the untreated GPMV (Figure 4J). The frequency of this occurrence under CC contact was statistically higher than silica and latex bead control (Figure 4K). Therefore, CC-mediated depletion of the membrane cholesterol is associated with a high probability of sudden rupture of vesicles in both live cells and GPMV, suggesting a signaling-independent, biophysical assault to the plasma membrane that result in a quick induction of necrotic cell death.

## Discussion

Besides apoptosis (Type I, caspase dependent) and autosis (Type II, ion channel dependent), cell death characterized by the rupture of plasma membrane (Type III) was believed to be a passive event, occurring in pathological states such as hypoxia (27, 51). The rapid release of cytosolic content was believed to dump intracellular “DAMPs” to the environment, causing inflammation. This notion of passive death has taken a dramatic turn in recent years. It has been found that under inhibition of caspase cascade, a typical apoptotic signal (such as TNFR ligation) can drive a different type of cell death, akin to necrosis (52). Mechanistically, TNFR1 signaling activates RIPK1 which subsequently activates RIPK3. RIPK3 phosphorylates MLKL, promoting the latter’s translocation to the plasma membrane (53). The helical structure of MLKL switches into a new conformation which forms uncontrolled cation channels on the membrane (54). This type of cell death differs from conventional necrosis in its sensitivity to RIPK1 inhibition (by necrostatin-1). Feng’s group found that Caspase1 activation by inflammasomes or direct LPS binding to Caspase 4/5/11 leads to GSDMD cleavage, sending its N-terminal fragment to be buried into the plasma membrane, creating a similar pore-forming structure (16). This type of cell death is termed pyroptosis due to the involvement of caspase activities. As these seminal findings delineate the precise mechanism of cell death upon membrane permeability changes, whether necrotic cell death can take place independent of these two pathways is not known. Except for physical damage associated with detergent treatment, ice crystal formation during freezing, and membrane piercing by sharp objects such as crystals, a physiological/pathologic situation whereby membrane rupture-mediated cell death is no longer a major concern to the mainstream immunologists. In this report, we propose that membrane lipid disruption, in this case, cholesterol depletion may be sufficient to induce membrane rupture “biophysically.” This finding, in turn, raises the possibility that small CC formed in the initiation stage of atherosclerosis may induce pro-inflammatory reactions simply *via* membrane destabilization in the absence of any known death pathway signaling.

The role of CC in atherosclerotic development is widely recognized (55). However, exactly how the smaller amounts of CC contribute to the initiation of the early pathology is a subject of study. While there is no doubt that CC can mediate NLRP3 activation that leads to IL-1β production (5), whether this pathway contributes to the disease development, however, is still debated (11). In our setting, the IL-1β production by phagocytes is irrelevant to the neutrophil infiltration, suggesting it is a relatively minor player. Our early paper provides a likely explanation (56). In an experimental gout model, the IL-1β produced by phagocytes signals through IL-1R on the parenchymal cells, opening a systematic feedback loop of IL-1β production (56). In other words, in the absence of involvement of non-hematopoietic cells, NLRP3 inflammasome activation by macrophages alone is insufficient for systemic inflammation. It is possible that for the formation of small CC in the artery walls, the signaling is not strong enough to reach a sustained inflammation, which may account for the lack of involvement of NLRP3 in atherosclerosis.

Previously, we proposed that in the absence of any receptor function, the ligation of plasma membrane lipids was sufficient to trigger lipid domain sorting and Syk-dependent phagocytic signaling (21). As the binding of CC to the plasma membrane exerts a force in the range of phagocytic events, it was unexpected that Syk was not required for such a strong force. The exact nature of this high binding affinity is unknown, although the exceedingly high binding force revealed in our AFM analysis of CC interaction with cholesterol-containing lipid domain may offer some clue. The extraction of cholesterol from the membrane may mimic the precursor condensation onto the crystalline surface in the typical growth of organic crystals (57), which may be translated into a force exerted on the plasma membrane. This notion, however, requires vigorous experimental verification, as alternative explanation may exist. Several recent papers took on the issue of solid particle-induced necrotic cell death, particularly with regard to neutrophil extracellular trap (NET) formation. It was found that nano particles, such as those of 10 nm diameter, triggered significant release of chromatin DNA that trapped those solid structures. Apparently, this NETosis may rely on cellular necrotic factors such as MLKL and RIPK1 (58), and may also require ROS production from NADPH oxidase complex (59). The question remains how those particles initiate their contact with the plasma membrane which can be regarded as the initial step for downstream cellular consequences (60). One proposal suggests that particle surface hydrophobicity may be critical for the membrane interactions. Simple decoration via surface PEGylation reduced the NETosis and DNA release, suggesting a loss of the ability of these particles to interact with phagocytes (60). Therefore, it is likely that a surface interaction, such as hydrophobicity-based attachment may be critical for CC to engage the membrane and contributes in some way to the cell death seen in our setting. CC-based cholesterol extraction then further weakens the plasma membrane. Whether hydrophobicity of particles plays a part in our system is, therefore, worthy of further analysis.

We do not understand why cholesterol depletion mediates the rapid “explosive” necrosis. The literature suggests the lipid homeostasis is critical for the plasma-membrane integrity and impacts various forms of programmed cell death (61). On the other hand, lipid disturbance can in general alter plasma-membrane permeability. In one proposal, with the increased amount of cholesterol, membrane orders increase, which prevents the pore formation particularly when the membrane is in an oxidative environment (62). Another possibility is that cholesterol in the membrane has a strong tendency to reduce the activities of several classes of ion channels, including inward-rectifying, voltage-gated and Ca^2+^ sensitive K^+^ channels, as well as voltage-gated Na^+^ and Ca^2+^ channels (63). The reduction of the cholesterol in the membrane may thus disrupt several ion and osmotic gradients that lead to membrane collapse. These, however, remain to be speculations until more refined experimental analyses. The implication of cholesterol depletion as a regulator for cardiovascular necrosis is at the moment difficult to prove experimentally, as such as “physical” depletion cannot be studied with a genetic model. Nevertheless, our work strongly suggests that clinical interventions via targeting IL-1β may be of limited value; the cholesterol control at the very early stage of atherosclerotic changes should be a more viable method for the prevention of cardiovascular diseases.

## Ethics Statement

All mouse experiment protocols were approved by the animal research committee of Tsinghua University.

## Author Contributions

FS performed all the experiments unless noted below. JC provided assistance in the atherosclerosis experiments. XM and YF provided help in cell culture, ELISA, and western blotting analyses. LY helped in cell death work. WZ helped in mouse husbandry. MA and TX provided technical support in AFM and lipid bilayer preparation. YS conceptualized the project, designed experiments, and wrote the manuscript.

## Conflict of Interest Statement

The authors declare that the research was conducted in the absence of any commercial or financial relationships that could be construed as a potential conflict of interest.

## Supplementary Material

Movie S1 CELL + CC: *GFP^tg^* BMDM were cultured on a glass slide. An AFM tip with cholesterol crystal was moved to touch the cell under constant height. GFP fluorescence of the cell was imaged every 20 s with a 488-nm laser in FITC channel. The time segment around the “explosion” was shown.

Movie S2 Giant plasma-membrane vesicle (GPMV) + CC: GFP-containing GPMV were harvested and “glued” to the bottom of glass disk with biotin and streptavidin. The rest was identical to Movie S1 in Supplementary Material except that the recording interval was 30 s.

Movie S3 Giant plasma-membrane vesicle + Empty tip: all procedures were identical to Movie S2 in Supplementary Material except AFM tip was replaced with an empty tip and all the recording frames were shown.

Movie S4 Giant plasma-membrane vesicle + Latex bead: all procedures were identical to Movie S3 in Supplementary Material except AFM tip was functionalized with a latex bead.

Movie S5 Giant plasma-membrane vesicle + Silica: all procedures were identical to Movie S3 in Supplementary Material except AFM tip was functionalized with a silica crystal.

## References

[1] Moore KJ Tabas I. Macrophages in the pathogenesis of atherosclerosis. Cell (2011) 145(3):341–55. 10.1016/j.cell.2011.04.005 21529710 PMC3111065

[2] Simons K Ikonen E. How cells handle cholesterol. Science (2000) 290(5497):1721–6. 10.1126/science.290.5497.1721 11099405

[3] Abela GS. Cholesterol crystals piercing the arterial plaque and intima trigger local and systemic inflammation. J Clin Lipidol (2010) 4(3):156–64. 10.1016/j.jacl.2010.03.003 21122648

[4] Abela GS Aziz K. Cholesterol crystals cause mechanical damage to biological membranes: a proposed mechanism of plaque rupture and erosion leading to arterial thrombosis. Clin Cardiol (2005) 28(9):413–20. 10.1002/clc.4960280906 16250264 PMC6654546

[5] Duewell P Kono H Rayner KJ Sirois CM Vladimer G Bauernfeind FG NLRP3 inflammasomes are required for atherogenesis and activated by cholesterol crystals. Nature (2010) 464(7293):1357–61. 10.1038/nature08938 20428172 PMC2946640

[6] Liu L Gardecki JA Nadkarni SK Toussaint JD Yagi Y Bouma BE Imaging the subcellular structure of human coronary atherosclerosis using micro-optical coherence tomography. Nat Med (2011) 17(8):1010–4. 10.1038/nm.2409 21743452 PMC3151347

[7] Lim RS Suhalim JL Miyazaki-Anzai S Miyazaki M Levi M Potma EO Identification of cholesterol crystals in plaques of atherosclerotic mice using hyperspectral CARS imaging. J Lipid Res (2011) 52(12):2177–86. 10.1194/jlr.M018077 21949051 PMC3220286

[8] Björkegren JL Hägg S Talukdar HA Foroughi Asl H Jain RK Cedergren C Plasma cholesterol-induced lesion networks activated before regression of early, mature, and advanced atherosclerosis. PLoS Genet (2014) 10(2):e1004201. 10.1371/journal.pgen.1004201 24586211 PMC3937269

[9] Hansson GK Hermansson A. The immune system in atherosclerosis. Nat Immunol (2011) 12(3):204–12. 10.1038/ni.2001 21321594

[10] Rajamäki K Lappalainen J Oörni K Välimäki E Matikainen S Kovanen PT Cholesterol crystals activate the NLRP3 inflammasome in human macrophages: a novel link between cholesterol metabolism and inflammation. PLoS One (2010) 5(7):e11765. 10.1371/journal.pone.0011765 20668705 PMC2909263

[11] Menu P Pellegrin M Aubert JF Bouzourene K Tardivel A Mazzolai L Atherosclerosis in ApoE-deficient mice progresses independently of the NLRP3 inflammasome. Cell Death Dis (2011) 2(3):e137. 10.1038/cddis.2011.18 21451572 PMC3101814

[12] Freigang S Ampenberger F Weiss A Kanneganti TD Iwakura Y Hersberger M Fatty acid-induced mitochondrial uncoupling elicits inflammasome-independent IL-1alpha and sterile vascular inflammation in atherosclerosis. Nat Immunol (2013) 14(10):1045–53. 10.1038/ni.2704 23995233

[13] Samstad EO Niyonzima N Nymo S Aune MH Ryan L Bakke SS Cholesterol crystals induce complement-dependent inflammasome activation and cytokine release. J Immunol (2014) 192(6):2837–45. 10.4049/jimmunol.1302484 24554772 PMC3985066

[14] Tabas I Consequences of cellular cholesterol accumulation: basic concepts and physiological implications. J Clin Invest (2002) 110(7):905–11. 10.1172/JCI0216452 12370266 PMC151158

[15] Mulay SR Desai J Kumar SV Eberhard JN Thomasova D Romoli S Cytotoxicity of crystals involves RIPK3-MLKL-mediated necroptosis. Nat Commun (2016) 7:10274. 10.1038/ncomms10274 26817517 PMC4738349

[16] Shi J Zhao Y Wang K Shi X Wang Y Huang H Cleavage of GSDMD by inflammatory caspases determines pyroptotic cell death. Nature (2015) 526(7575):660–5. 10.1038/nature15514 26375003

[17] Zhivotovsky B Orrenius S. Calcium and cell death mechanisms: a perspective from the cell death community. Cell Calcium (2011) 50(3):211–21. 10.1016/j.ceca.2011.03.003 21459443

[18] Liu X Van Vleet T Schnellmann RG. The role of calpain in oncotic cell death. Annu Rev Pharmacol Toxicol (2004) 44:349–70. 10.1146/annurev.pharmtox.44.101802.121804 14744250

[19] Desrosiers MD Cembrola KM Fakir MJ Stephens LA Jama FM Shameli A Adenosine deamination sustains dendritic cell activation in inflammation. J Immunol (2007) 179(3):1884–92. 10.4049/jimmunol.179.3.1884 17641055

[20] Kobayashi M Inoue K Warabi E Minami T Kodama T. A simple method of isolating mouse aortic endothelial cells. J Atheroscler Thromb (2005) 12(3):138–42. 10.5551/jat.12.138 16020913

[21] Ng G Sharma K Ward SM Desrosiers MD Stephens LA Schoel WM Receptor-independent, direct membrane binding leads to cell-surface lipid sorting and Syk kinase activation in dendritic cells. Immunity (2008) 29(5):807–18. 10.1016/j.immuni.2008.09.013 18993083 PMC2642965

[22] Beattie JH Duthie SJ Kwun IS Ha TY Gordon MJ. Rapid quantification of aortic lesions in apoE(-/-) mice. J Vasc Res (2009) 46(4):347–52. 10.1159/000189795 19142014

[23] Flach TL Ng G Hari A Desrosiers MD Zhang P Ward SM Alum interaction with dendritic cell membrane lipids is essential for its adjuvanticity. Nat Med (2011) 17(4):479–87. 10.1038/nm.2306 21399646

[24] Hari A Zhang Y Tu Z Detampel P Stenner M Ganguly A Activation of NLRP3 inflammasome by crystalline structures via cell surface contact. Sci Rep (2014) 4:7281. 10.1038/srep07281 25445147 PMC4250918

[25] Sezgin E Kaiser HJ Baumgart T Schwille P Simons K Levental I. Elucidating membrane structure and protein behavior using giant plasma membrane vesicles. Nat Protoc (2012) 7(6):1042–51. 10.1038/nprot.2012.059 22555243

[26] Unsay JD Cosentino K Garcia-Saez AJ Atomic force microscopy imaging and force spectroscopy of supported lipid bilayers. J Vis Exp (2015) 101:e52867 10.3791/52867 PMC4545161 26273958

[27] Golstein P Kroemer G. Cell death by necrosis: towards a molecular definition. Trends Biochem Sci (2007) 32(1):37–43. 10.1016/j.tibs.2006.11.001 17141506

[28] Hagar JA Powell DA Aachoui Y Ernst RK Miao EA. Cytoplasmic LPS activates caspase-11: implications in TLR4-independent endotoxic shock. Science (2013) 341(6151):1250–3. 10.1126/science.1240988 24031018 PMC3931427

[29] Franchi L Eigenbrod T Muñoz-Planillo R Nuñez G. The inflammasome: a caspase-1-activation platform that regulates immune responses and disease pathogenesis. Nat Immunol (2009) 10(3):241–7. 10.1038/ni.1703 19221555 PMC2820724

[30] Kayagaki N Warming S Lamkanfi M Vande Walle L Louie S Dong J Non-canonical inflammasome activation targets caspase-11. Nature (2011) 479(7371):117–21. 10.1038/nature10558 22002608

[31] Muñoz-Planillo R Kuffa P Martínez-Colón G Smith BL Rajendiran TM Núñez G K+ efflux is the common trigger of NLRP3 inflammasome activation by bacterial toxins and particulate matter. Immunity (2013) 38(6):1142–53. 10.1016/j.immuni.2013.05.016 23809161 PMC3730833

[32] Cassel SL Eisenbarth SC Iyer SS Sadler JJ Colegio OR Tephly LA The Nalp3 inflammasome is essential for the development of silicosis. Proc Natl Acad Sci U S A (2008) 105(26):9035–40. 10.1073/pnas.0803933105 18577586 PMC2449360

[33] Eisenbarth SC Colegio OR O’Connor W Sutterwala FS Flavell RA. Crucial role for the Nalp3 inflammasome in the immunostimulatory properties of aluminium adjuvants. Nature (2008) 453(7198):1122–6. 10.1038/nature06939 18496530 PMC4804622

[34] Mariathasan S Weiss DS Newton K McBride J O’Rourke K Roose-Girma M Cryopyrin activates the inflammasome in response to toxins and ATP. Nature (2006) 440(7081):228–32. 10.1038/nature04515 16407890

[35] Zhang SH Reddick RL Piedrahita JA Maeda N. Spontaneous hypercholesterolemia and arterial lesions in mice lacking apolipoprotein E. Science (1992) 258(5081):468–71. 10.1126/science.1411543 1411543

[36] Rock KL Kono H. The inflammatory response to cell death. Annu Rev Pathol (2008) 3:99–126. 10.1146/annurev.pathmechdis.3.121806.151456 18039143 PMC3094097

[37] Marichal T Ohata K Bedoret D Mesnil C Sabatel C Kobiyama K DNA released from dying host cells mediates aluminum adjuvant activity. Nat Med (2011) 17(8):996–1002. 10.1038/nm.2403 21765404

[38] Galluzzi L Vitale I Abrams JM Alnemri ES Baehrecke EH Blagosklonny MV Molecular definitions of cell death subroutines: recommendations of the nomenclature committee on cell death 2012. Cell Death Differ (2012) 19(1):107–20. 10.1038/cdd.2011.96 21760595 PMC3252826

[39] Han J Zhong C-Q Zhang D-W. Programmed necrosis: backup to and competitor with apoptosis in the immune system. Nat Immunol (2011) 12(12):1143–9. 10.1038/ni.2159 22089220

[40] Wu J Huang Z Ren J Zhang Z He P Li Y Mlkl knockout mice demonstrate the indispensable role of Mlkl in necroptosis. Cell Res (2013) 23(8):994–1006. 10.1038/cr.2013.91 23835476 PMC3731568

[41] Ofengeim D Yuan JY. Regulation of RIP1 kinase signalling at the crossroads of inflammation and cell death. Nat Rev Mol Cell Biol (2013) 14(11):727–36. 10.1038/nrm3683 24129419

[42] Philip NH Dillon CP Snyder AG Fitzgerald P Wynosky-Dolfi MA Zwack EE Caspase-8 mediates caspase-1 processing and innate immune defense in response to bacterial blockade of NF-κB and MAPK signaling. Proc Natl Acad Sci U S A (2014) 111(20):7385–90. 10.1073/pnas.1403252111 24799700 PMC4034241

[43] Zhong Z Zhai Y Liang S Mori Y Han R Sutterwala FS TRPM2 links oxidative stress to NLRP3 inflammasome activation. Nat Commun (2013) 4:1611. 10.1038/ncomms2608 23511475 PMC3605705

[44] Chen J Ganguly A Mucsi AD Meng J Yan J Detampel P Strong adhesion by regulatory T cells induces dendritic cell cytoskeletal polarization and contact-dependent lethargy. J Exp Med (2017) 214(2):327–38. 10.1084/jem.20160620 28082358 PMC5294852

[45] Rensen S Doevendans P Van Eys G. Regulation and characteristics of vascular smooth muscle cell phenotypic diversity. Neth Heart J (2007) 15(3):100–8. 10.1007/BF03085963 17612668 PMC1847757

[46] Flannagan RS Jaumouillé V Grinstein S The cell biology of phagocytosis. Annu Rev Pathol (2012) 7(1):61–98. 10.1146/annurev-pathol-011811-132445 21910624

[47] Jaumouillé V Farkash Y Jaqaman K Das R Lowell CA Grinstein S Actin cytoskeleton reorganization by syk regulates fcγ receptor responsiveness by increasing its lateral mobility and clustering. Dev Cell (2014) 29(5):534–46. 10.1016/j.devcel.2014.04.031 24914558 PMC4083245

[48] Sebzda E Hibbard C Sweeney S Abtahian F Bezman N Clemens G Syk and Slp-76 mutant mice reveal a cell-autonomous hematopoietic cell contribution to vascular development. Dev Cell (2006) 11(3):349–61. 10.1016/j.devcel.2006.07.007 16950126

[49] Connell SD Heath G Olmsted PD Kisil A. Critical point fluctuations in supported lipid membranes. Faraday Discuss (2013) 161:91–111; discussion 113–50. 10.1039/C2FD20119D 23805740

[50] Kellner-Weibel G Yancey PG Jerome WG Walser T Mason RP Phillips MC Crystallization of free cholesterol in model macrophage foam cells. Arterioscler Thromb Vasc Biol (1999) 19(8):1891–8. 10.1161/01.ATV.19.8.1891 10446067

[51] Zong WX Thompson CB Necrotic death as a cell fate. Genes Dev (2006) 20(1):1–15. 10.1101/gad.1376506 16391229

[52] Degterev A Huang Z Boyce M Li Y Jagtap P Mizushima N Chemical inhibitor of nonapoptotic cell death with therapeutic potential for ischemic brain injury. Nat Chem Biol (2005) 1(2):112–9. 10.1038/nchembio711 16408008

[53] Sun L Wang H Wang Z He S Chen S Liao D Mixed lineage kinase domain-like protein mediates necrosis signaling downstream of RIP3 kinase. Cell (2012) 148(1–2):213–27. 10.1016/j.cell.2011.11.031 22265413

[54] Xia B Fang S Chen X Hu H Chen P Wang H MLKL forms cation channels. Cell Res (2016) 26(5):517–28. 10.1038/cr.2016.26 27033670 PMC4856759

[55] Grebe A Latz E. Cholesterol crystals and inflammation. Curr Rheumatol Rep (2013) 15(3):313. 10.1007/s11926-012-0313-z 23412688 PMC3623938

[56] Chen CJ Shi Y Hearn A Fitzgerald K Golenbock D Reed G MyD88-dependent IL-1 receptor signaling is essential for gouty inflammation stimulated by monosodium urate crystals. J Clin Invest (2006) 116(8):2262–71. 10.1172/JCI28075 16886064 PMC1523415

[57] Jiang Y Kellermeier M Gebaue D Lu Z Rosenberg R Moise A Growth of organic crystals via attachment and transformation of nanoscopic precursors. Nat Commun (2017) 8:15933. 10.1038/ncomms15933 28635962 PMC5482053

[58] Desai J Foresto-Neto O Honarpisheh M Steiger S Nakazawa D Popper B Particles of different sizes and shapes induce neutrophil necroptosis followed by the release of neutrophil extracellular trap-like chromatin. Sci Rep (2017) 7(1):15003. 10.1038/s41598-017-15106-0 29101355 PMC5670218

[59] Biermann MH Podolska MJ Knopf J Reinwald C Weidner D Maueröder C Oxidative burst-dependent NETosis is implicated in the resolution of necrosis-associated sterile inflammation. Front Immunol (2016) 7:557. 10.3389/fimmu.2016.00557 27990145 PMC5131011

[60] Muñoz LE Bilyy R Biermann MH Kienhöfer D Maueröder C Hahn J Nanoparticles size-dependently initiate self-limiting NETosis-driven inflammation. Proc Natl Acad Sci U S A (2016) 113(40):E5856–65. 10.1073/pnas.1602230113 27647892 PMC5056044

[61] Agmon E Stockwell BR. Lipid homeostasis and regulated cell death. Curr Opin Chem Biol (2017) 39:83–9. 10.1016/j.cbpa.2017.06.002 28645028 PMC5581689

[62] Van der Paal J Neyts EC Verlackt CCW Bogaerts A. Effect of lipid peroxidation on membrane permeability of cancer and normal cells subjected to oxidative stress. Chem Sci (2016) 7(1):489–98. 10.1039/c5sc02311d 28791102 PMC5518669

[63] Levitan I Fang Y Rosenhouse-Dantsker A Romanenko V Cholesterol and ion channels. Subcell Biochem (2010) 51:509–49. 10.1007/978-90-481-8622-8_19 20213557 PMC2895485

