# Supplementary material for: Cholesterol Crystal-Mediated Inflammation Is Driven by Plasma Membrane Destabilization
Source: Front Immunol. 2018 May 29;9:1163. doi: 10.3389/fimmu.2018.01163 (PMC5986904; doi:10.3389/fimmu.2018.01163)
Supplement: Supplementary file 6 [file image_1.PDF]

# Supplemental data

## Supp Fig 1

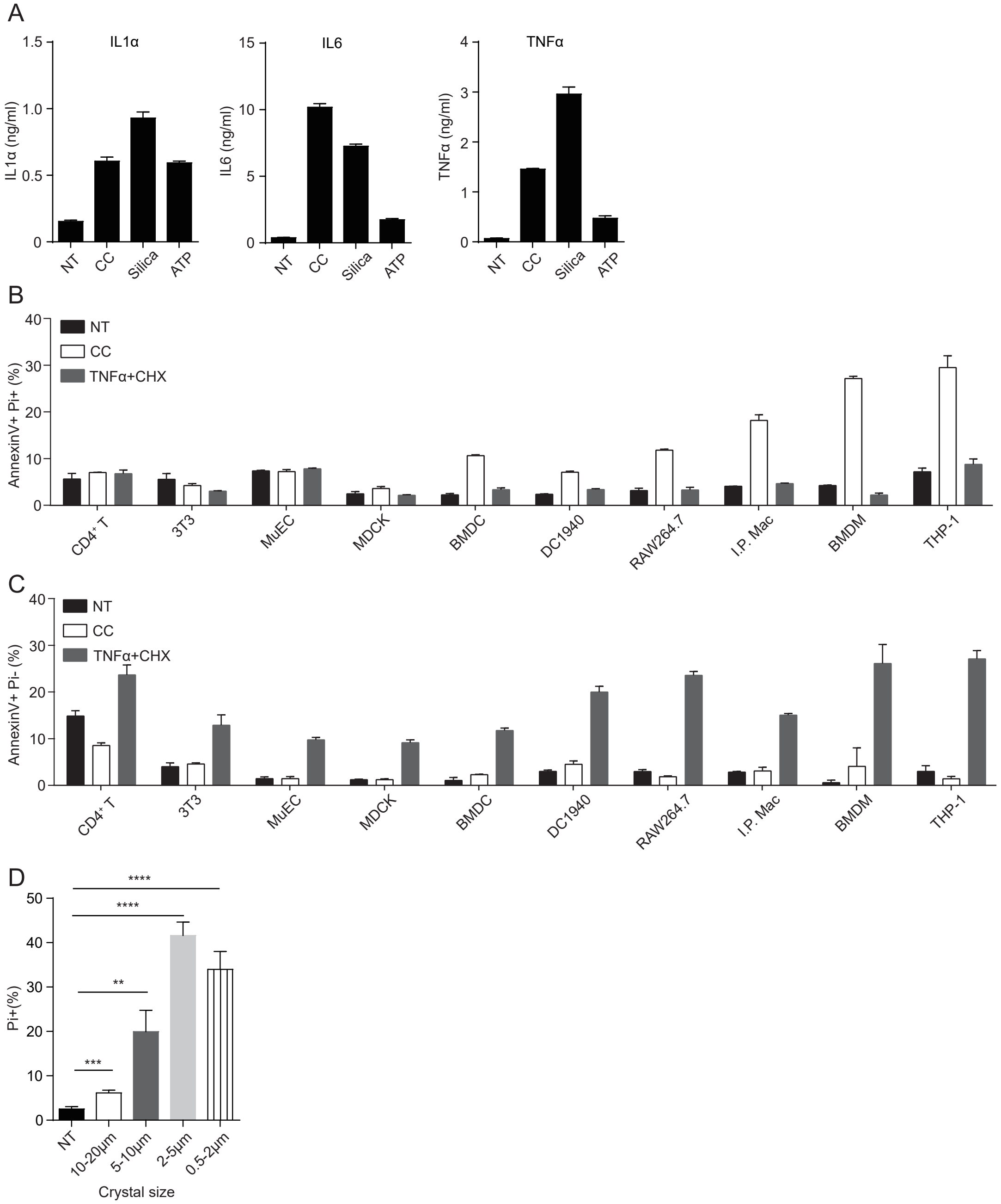

Supp Fig 1. A. BMDM were treated as Fig. 1C except cells had not been primed. Cytokines in the supernatants were detected with ELISA. n=4. N=3. B&C. Different type of cells were treated with CC (500 μg/ml) or TNFα+CHX (TNFα: 100 ng/ml, CHX: 50 μg/ml) for 2 hr before Annexin V and Pi staining. Samples were read with flowcytometry. Annexin V+ PI+ or Annexin V+ PI- were shown. n=2. N=3. D. BMDM were treated with different sizes of CC for 2 hr before PI staining. n=12. N=3.
