# Supplementary material for: Cholesterol Crystal-Mediated Inflammation Is Driven by Plasma Membrane Destabilization
Source: Front Immunol. 2018 May 29;9:1163. doi: 10.3389/fimmu.2018.01163 (PMC5986904; doi:10.3389/fimmu.2018.01163)
Supplement: Supplementary file 7 [file image_2.PDF]

Supplemental data

Supp Fig 2

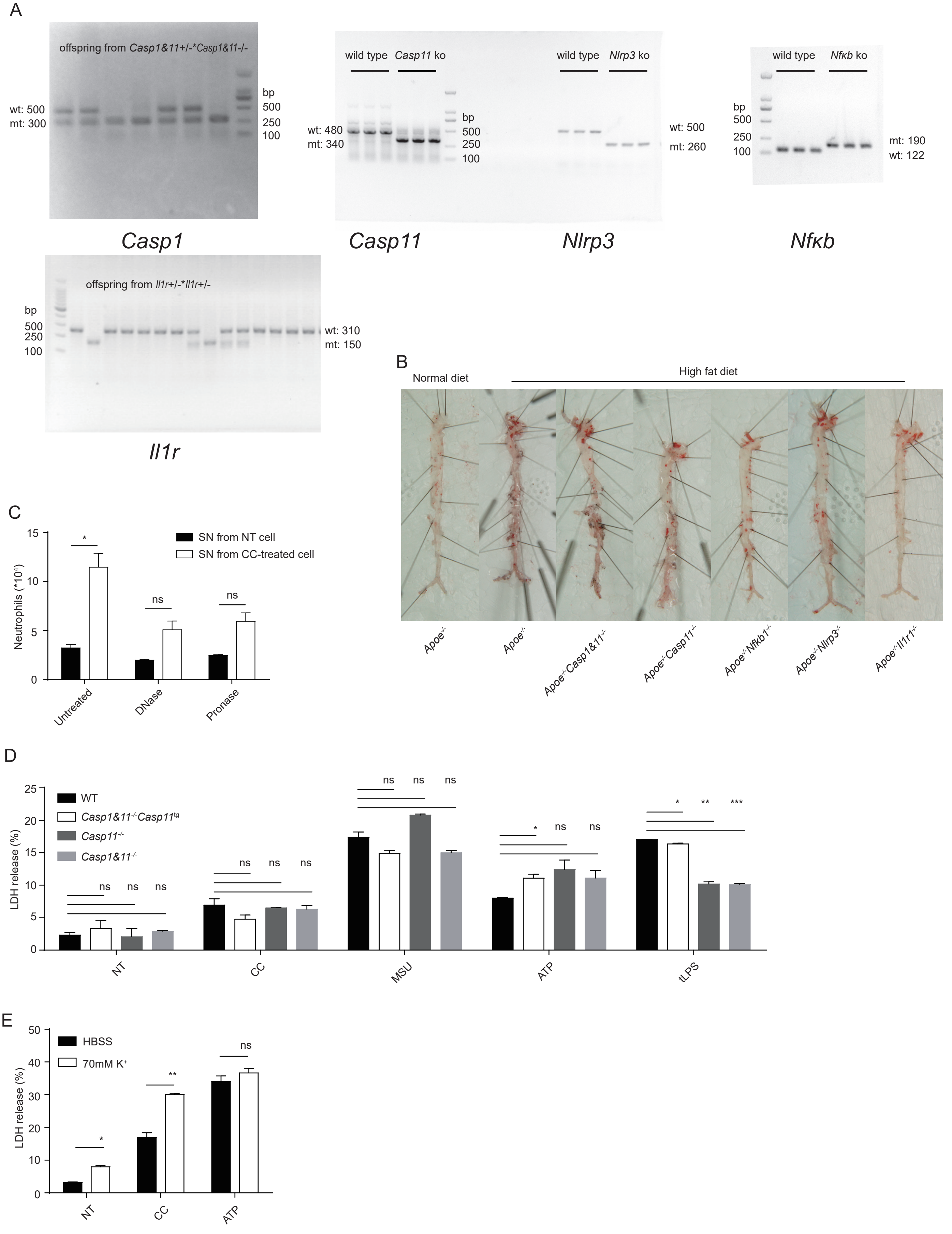

Supp Fig 2. A. Genotypes of gene deficient mice in Fig 2 were verified by PCR with primers listed in the methods. Genotype with mt band alone indicates homozygote mutation. B. The aortas of Fig. 2E were opened *en face* for analysis. The plaque surface area values were taken from these samples. C. Supernatants were collected as in Fig1B then treated with DNase I (3 U/ml) or Pronase (10 ng/ml) in 37 ℃ for 4 hr. The lefts were same as Fig1B. n=2, N=3. D. BMDM were primed and then treated as in Fig. 1C for 6 hr (MSU: 100 μg/ml). Supernatants were collected and cell death was determined by LDH assay. n=3. N=3. E. BMDM were primed as in Fig. 1C then the medium was replaced with HBSS or 70mM K^+ HBSS. 15 min after, cells were treated as in Fig. 1C for 12 hr then cell death was determined by LDH assay. n=3. N=3.
