# Supplementary material for: Cholesterol Crystal-Mediated Inflammation Is Driven by Plasma Membrane Destabilization
Source: Front Immunol. 2018 May 29;9:1163. doi: 10.3389/fimmu.2018.01163 (PMC5986904; doi:10.3389/fimmu.2018.01163)
Supplement: Supplementary file 8 [file image_3.PDF]

Supplemental data

Supp Fig 3

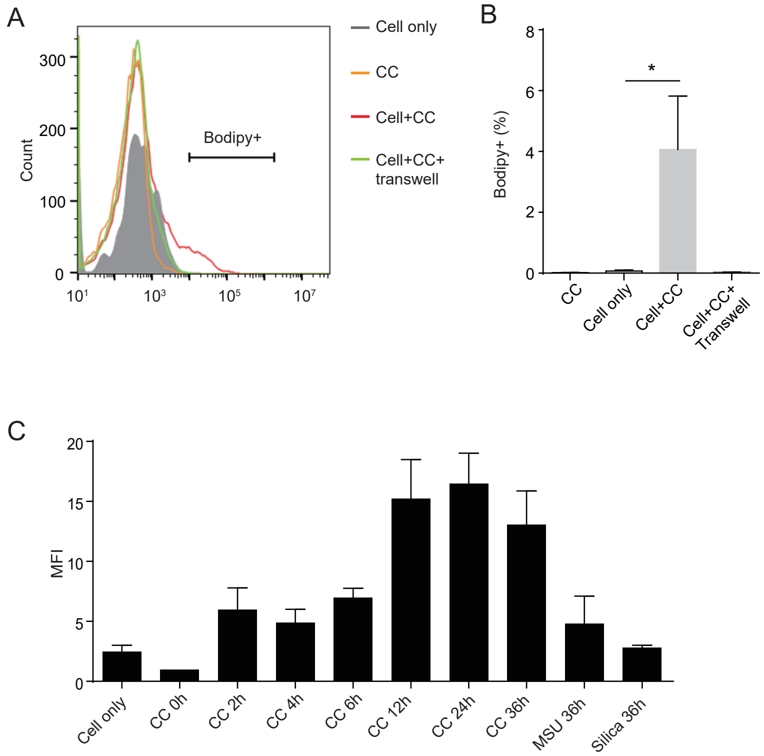

Supp Fig 3A&B. Cells were labeled with Bodipy-cholesterol by MβCD. CC (200 μg/ml) were added to the cells directly or separated with a transwell plate (0.5 μm). 12 hr later, crystals were analyzed by FACS. n=3. A. Histograph of bodipy fluorescence in each group. B. bodipy+ ratio in each group. n=3. N=3.

Supp Fig 3C. Mean fluorescence of crystals in Fig. 4D was measured by ImageJ. n=6.
